# Testosterone Administration Decreases Generosity in the Ultimatum Game

**DOI:** 10.1371/journal.pone.0008330

**Published:** 2009-12-16

**Authors:** Paul J. Zak, Robert Kurzban, Sheila Ahmadi, Ronald S. Swerdloff, Jang Park, Levan Efremidze, Karen Redwine, Karla Morgan, William Matzner

**Affiliations:** 1 Center for Neuroeconomics Studies, Claremont Graduate University, Claremont, California, United States of America; 2 University of Pennsylvania, Philadelphia, Pennsylvania, United States of America; 3 Department of Endocrinology, University of California Los Angeles, Los Angeles, California, United States of America; 4 Division of Endocrinology, Harbor University of California Los Angeles Medical Center, Torrance, California, United States of America; 5 Department of Economics, Whitworth University, Spokane, Washington, United States of America; University of Groningen, The Netherlands

## Abstract

How do human beings decide when to be selfish or selfless? In this study, we gave testosterone to 25 men to establish its impact on prosocial behaviors in a double-blind within-subjects design. We also confirmed participants' testosterone levels before and after treatment through blood draws. Using the Ultimatum Game from behavioral economics, we find that men with artificially raised T, compared to themselves on placebo, were 27% less generous towards strangers with money they controlled (95% CI placebo: (1.70, 2.72); 95% CI T: (.98, 2.30)). This effect scales with a man's level of total-, free-, and dihydro-testosterone (DHT). Men in the lowest decile of DHT were 560% more generous than men in the highest decile of DHT. We also found that men with elevated testosterone were more likely to use their own money punish those who were ungenerous toward them. Our results continue to hold after controlling for altruism. We conclude that elevated testosterone causes men to behave antisocially.

## Introduction

Human beings are both prosocial and self-serving, often exhibiting both behaviors in a short period of time. The neurologic foundations for prosociality are just beginning to be examined [1]–[4], but the mechanisms that cause a shift from selfless to selfish have not been characterized.

There is an extensive literature associating male aggressive and antisocial behaviors with elevated testosterone (T) [5], [6]. Yet, T is not the most obvious candidate promoting selfishness; the recent multi-billion dollar donations to charity by Bill Gates and Warren Buffett reveal that males with significant resources may be generous. Studies in monkeys show that when beta males become alphas, both T and serotonin rise while cortisol falls [7], [8]. Alpha males have been observed sharing resources, but this is typically strategic, for example, to sustain a supporting coalition [9], [10]. Yet, alpha males, unlike lower ranking members of a social group, may have less need to be generous towards others. Correlational studies of salivary T in humans have found that high T males are more likely to have physical altercations, divorce more often, spend less time with their children, engage in competitions of all types, have more sexual partners, face learning disabilities, and lose their jobs more often [11], [12] suggesting that high T men may behave differently than other men. A recent study found that high T males are more likely to reject stingy offers in the Ultimatum Game [13], but whether high T is the cause or the effect of a low offer is unclear; low offers in a related task called the trust game have been associated with a rise in serum dihydrotestosterone [14]. More generally, high T males appear to be more aggressive and less prosocial [15].

These correlations should be viewed with caution as T is highly dependent on a variety of environmental conditions [11]. For example, winning a chess match will raise T, and watching one's team lose a soccer game on TV will cause T to fall [16], [17]. The inability to control experimental subjects' behaviors before they enter the lab, and the high degree of variability in basal T indicate that correlational studies can only be considered provisional findings [18]. In addition, salivary testosterone assays, while convenient, have measurement problems, including the effect of foreign substances such as gum to facilitate salivation and contamination with blood due to microtrauma. Further, there is only a moderate correlation between T measured in saliva and blood serum [19].

Critiquing correlational studies of T and behavior, O'Carroll wrote that “Definitive evidence is likely to come from placebo-controlled, double-blind experiments in which circulating T levels are manipulated and appropriately reliable and sensitive assays of behaviour are taken.” [18]. Manipulating T produces direct causal evidence directly relating to T to behavior, and this is precisely the approach we take here. Yet, hormone manipulation is rare in the nonclinical literature. Studies that infused moderate supraphysiologic doses of T into eugonadal males have found little effect on anger or mood [20]–[22]; mood effects occur only for very high doses of T. A very small study (N = 6) showed that men who had their T raised for six weeks, compared to themselves on placebo, were more likely to respond in kind to a perceived provocation (though actually fictitious) in which they were made to lose a small amount of money by another person [23].

In the present study, we manipulated T in healthy eugonadal men in a double-blind, cross-over study to examine the effects of T on social behaviors. Using a neuroeconomics paradigm [24], participants made a set of decisions involving money. We hypothesized that T would cause men to behave less generously towards strangers. These tasks also allowed us to measure the incidence of punishment of those who violate an implicit social norm of generosity. We hypothesized that participants given T would be more likely to punish those making ungenerous monetary offers to them.

## Materials and Methods

Forty-eight male students were recruited for this double-blind cross-over experiment. The mean age of participants was 20.8 years old (SD = 2.2), and the sample was ethnically diverse (Asian 44%, Caucasian 36%, Hispanic 8%, Other/no data 12%). Only male participants were recruited because the US Food and Drug Administration has only approved testosterone treatment for men, and men were likely to be more reactive behaviorally to its effects [25]. Twenty-five participants completed the entire experiment and are included in our analyses. All participants gave written informed consent for the study, with study phases (testosterone or placebo) separated by six to 12 weeks depending on which sessions participants were in. In every session, approximately one-half of the participants received testosterone and the other half were given the placebo. Session sizes varied from four to eight participants. The experiment was approved by the Institutional Review Boards of UCLA and Claremont Graduate University.

For every session, participants arrived at 4 pm and were interviewed by a licensed medical doctor (S.A.) for possible contraindications for T administration. Exclusion criteria included significant medical or psychiatric illness, medications that interact with T, and drug or alcohol abuse. After medical screening and consent, participants had 28 ml of blood draw from an antecubital vein. Next, participants were led to a semi-private booth, asked to remove their shirts, and were given a colorless hydroalcoholic gel containing either 10 g of Androgel® (1% testosterone gel) or an inert substance. Participants were instructed and observed spreading the gel on their shoulders and upper back following the Androgel® instructions. No adverse events were reported. On debriefing, participants reported that they did not know which substance they had been given.

Following published pharmacokinetics [26] on peak levels of T, participants returned to the lab 16 hours after administration for a second blood draw, to answer survey questions, and make a series of decisions involving money. The blood draw for the 8am session established how much higher participants' T levels were after Androgel® administration. After the second blood draw, participants completed questionnaires by computer using a random alphanumeric code as their only identifier. The questionnaires measured demographic, social, and psychological traits. These included Experiences in Close Relationships-Revised (ECR-R) [27] that measures attachment styles, the Interpersonal Reactivity Index (IRI; 28, 29), which measures dispositional empathy, Affective Intensity Measure that addresses emotional responses (AIM) [30], an Anger Inventory [4], and the Personal Reaction Inventory (PRI)[31] that measures social behaviors.

Participants next made decisions in two tasks, the Ultimatum game (UG) and the Dictator Game (DG), that involve money and other people. All decisions were made by computer in partitioned stations and without communicating to others in the experiment. In both the UG and DG, participants were randomly assigned by computer to dyads. Within a dyad, there was a decision-maker 1 (DM1) and decision-maker 2 (DM2). In all tasks, both DMs received extensive and identical instructions regarding how their decisions and those of the other DM in the dyad would affect how much money each could make. The UG and DG are standard tasks in experimental economics and neutral language in the instructions was used throughout.

**Figure 1:**
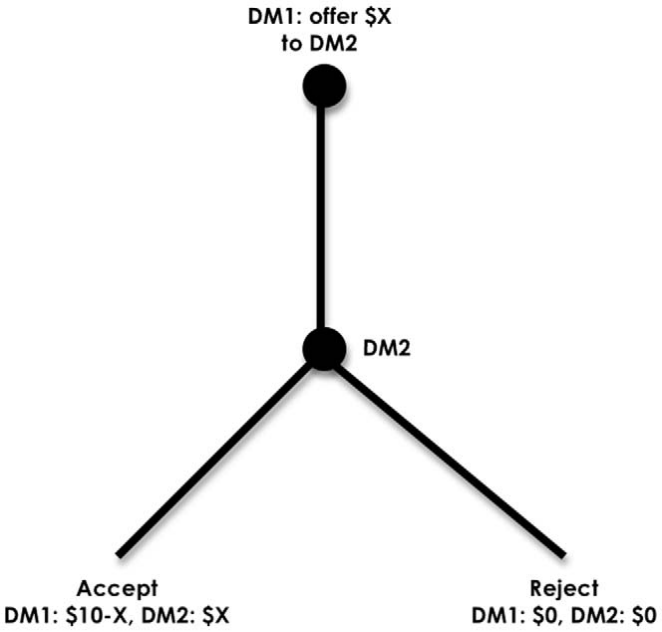
In the Ultimatum Game, Decision-Maker 1 (DM1) is endowed with $10 and DM2 has no endowment. DM1 proposes a split of his endowment to DM2 that DM2 can either accept or reject. Accepted proposals are paid to both DMs while rejected proposals cause both DMs to receive nothing. All participants made proposals as DM1s and as DM2s were asked to state their minimum acceptable proposal to elicit their punishment thresholds. After making decisions as DM1 and DM2, the roles were randomized to determine earnings. Both DMs were fully and identically instructed in this task. The subgame perfect equilibrium is for DM1 to offer $1 and for DM2 to accept this.

In the UG, DM1 was endowed with $10 while DM2 had nothing. After instruction, DM1 was prompted by computer to propose a split of this money to DM2. DM2 could either accept the proposal and then the money would be paid, or he could reject the proposal and both DM2s would get nothing (Fig. 1). All participants were asked to make proposals as DM1s and to identify their minimum their acceptable offers as DM2s. At the end of the experiment, payment was determined by randomly assigning each person to the role of DM1 or DM2 for each decision.

By using the “strategy method” in which participants make both proposals at DM1s and state their punishment threshold as DM2s [32], we are able to perform within-subjects comparisons of behavior. Participants were fully instructed that all decisions were “live” in that after making decisions, DM1 to DM2 pairings would be made that determined their earnings. Participants appeared to understand this because there was variation in UG choices across rounds (average within-subjects SD of: proposals $0.68; punishment threshold $1.19; and generosity $1.55). There is agreement in experimental economics that using the strategy method with payments produces very similar data to real-time dyadic matching.

Following a related study, the UG was used to measure generosity [33]. A generous offer is defined as the difference between the DM1 proposal and the participant's own minimum acceptable offer as DM2. Proposals of exactly the minimum acceptable amount are not generous because they do not demonstrate “liberality in giving” or offering more than another person expects or needs.

The UG can also be used to measure the willingness by individuals to engage in costly punishment of stingy offers or for violations of implicit sharing norms. In Western countries, offers less than 30% of DM1's endowment are nearly always rejected [32]. Stingy offers to DM2s in the UG have been shown to provoke anterior insula activity [34] suggesting that low offers are rejected due to a sense of disgust. A high minimum acceptable offer therefore punishes DM1 for stingy offer but at a cost to DM2.

The DG was included as a control. In this task, participants were also randomly put into dyads in which DM1 had $10 and DM2 had zero. After instruction, DM1 was asked to make a unilateral offer of some of his endowment to the DM2 in the dyad. DM2 had no choice to make. Money transferred in the DG is thought to measure altruism [32]. Participants made decisions in the UG and DG four times with random rematching to other DMs each round. Participants were instructed that they would make four one-shot decisions. This approach was used to expose fewer participants to the effects of drugs following a protocol we have previously used with oxytocin infusion [35]. At the end of the experiment, participants were paid their earnings privately by a lab administrator. There was no deception of any kind.

Although the data are not normally distributed (Shapiro-Wilk test p = .001), the large sample size and paired data with a central moment and kurtosis indicate that t-tests are appropriate for the analysis [36]–[38].

## Results

### Testosterone

First, we established that for those receiving the placebo that T levels did not rise overnight. We assayed total, free, and dihydrotestosterone (DHT) to fully characterize the androgenic state of participants. All assays were performed by Yerkes Biomarkers Core using kits from Diagnostic Systems Laboratories (Webster, TX). CVs for assays where within acceptable ranges, DHT (Inter-assay: 7.32% at 118.03 pg/ml, n = 4, Intra-assay: 7.17% at 624.30 pg/ml, n = 4,); Total T (Inter-assay: 1.55% at 3.04 pg/ml, n = 2, Intra-assay: 1.60% at 23.87 pg/ml, n = 2); Free T (Inter-assay: 5.95% at 0.68 ng/ml, 4.14% at 5.67 ng/ml, Intra-assay: 6.3%, at 0.86 ng/ml, n = 6.

**Figure 2:**
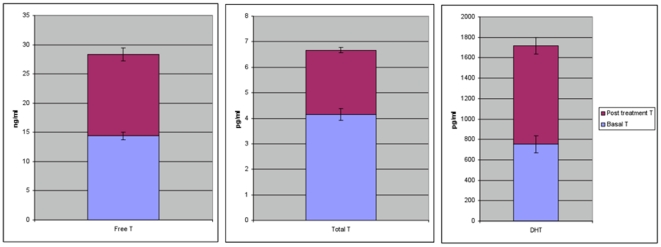
Values of total T, free T, and DHT before and after Androgel® treatment; all differences p<1E-6. The blue bar is basal T and the blue plus the red bar is the post-treatment T value (and SE bars). T in every treated subject was higher than baseline. The highest level of total T after treatment was 10.32 pg/ml or a 170% change from baseline; the smallest change in total T was 0.31 pg/ml or a 7% change.

For men receiving placebo, average T values before infusion and 16 hours later were unchanged (total T: 4.3 pg/ml (SD .92), 4.0 pg/ml (SD .63) paired t-test p = .84; free T: 15.1 ng/ml (SD 3.34), ng/ml (SD 3.08), paired t-test p = .64; DHT: 704.2 pg/ml (SD = 228.6), 809.1 pg/ml (SD = 267.9), paired t-test p = .99). We next assessed whether T was higher after 10 g of Androgel® treatment. Average T levels prior to Androgel® treatment were total T: 4.2 pg/ml (SD .93); free T: 14.4 ng/ml (SD 3.52), and DHT: 753.3 pg/ml (SD 413.39). Sixteen hours after Androgel® treatment, total T was 60% higher, free T was 97% higher, and DHT was 128% higher (SDs, 1.85; 10.79; 736.48); Fig. 2. All of these changes were greater than zero for p<1E-6 (two-tailed paired t-tests, N = 50). In addition, because T was elevated in every participant given Androgel® compared to himself, no one was excluded from the analyses.

### Behavior

**Figure 3:**
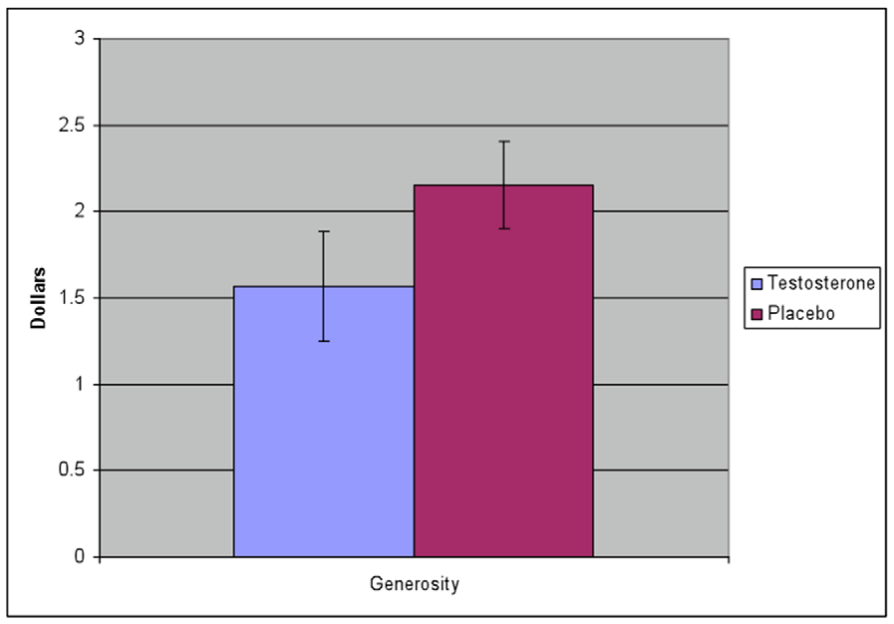
Generosity (UG offer - UG punishment threshold) by participants on placebo was $2.15 compared to $1.57 when the same individuals were given Androgel®, a 27% reduction (p = .035; bars in graph are SEs). More participants on Androgel® relative to placebo showed negative generosity by setting a punishment threshold above than their own offer to DM2 (9.6% vs. 2.9%).

Average DM1 proposals in the UG were 9% lower for men on T compared to themselves on placebo (T: $4.63, Placebo: $5.08, one-tailed paired t-test, N = 200, p = .001). At the same time, the DM2 rejection threshold was 5% higher on T versus placebo though the difference was not significant (T: $3.05, Placebo: $2.92, two-tailed paired t-test, N = 200, p = .61). T infusion did affect the amount of negative generosity (proposals<rejection threshold), with 9.6% of participants on Androgel® rejecting their own proposals compared to 2.9% rejections for participants on placebo (p = .046, two-tailed t test). Consistent with our primary hypothesis, generosity (proposals - rejection threshold) by men on T compared to themselves on placebo was 27% lower (T: $1.57, Placebo: $2.15, one-tailed paired t-test, N = 200, p = .035; Fig. 3).

**Figure 4:**
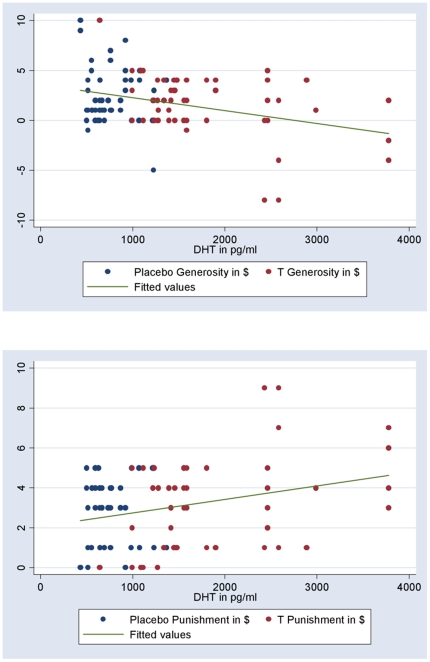
Scale effect of T on generosity. T levels and generosity for those on placebo are shown in blue, generosity for those on Androgel® are shown in red. (A) The reduction of generosity for those on Androgel® positively scales with levels of total T, free T and DHT; the relationship for DHT is shown (r = −0.3063, p = 0.0001). Men in the lowest decile of DHT had average generosity of $3.65 compared to generosity of $0.55 for men in the highest decile of DHT (85% lower). (B) The punishment threshold also scales with a man's level of total T, free T and DHT; the relationship for DHT is shown (r = 0.2284, p = 0.0011). Men in the lowest decile of DHT had average punishment threshold of $2.15 compared to a punishment threshold of $4.00 for men in the top decile of DHT (86% higher).

To confirm our results, we ran a random-effects GLM of generosity and a T indicator variable for DM1 offers, DM2 rejection threshold, and generosity. Our basic findings showing that T makes men less generous continue to hold (DM1 offers: coeff. = −.46>0, p = .0001; generosity: coeff. = −.57>0, p = .048; rejection threshold: coeff. = .11>0, p = .64). Next, we examined if there was a parametric relationship between T, generosity, and punishment. Testing all three measures of T (free, total, and DHT), we found that greater T was associated with less generosity and an increased desire to punish those making stingy offers. For generosity we found highly significant correlations with total T (r = −0.25, two-tailed t test, N = 200 for this and subsequent tests, p = 0.0004), free T (r = −0.1908, p = 0.0068), and DHT (r = −0.3063, p = 0.0001); see Fig. 4A. Significant correlations were also found for the rejection threshold and total T (r = 0.1937, p = 0.0060), free T (r = 0.1529, p = 0.0306), and DHT (r = 0.2284, p = 0.0011); see Fig. 4B.

Behavioral studies of strategic economic games have found learning effects from repeat play [32]. As a result, we tested if behavior differed when a participant received Androgel® or placebo on the first phase of the experiment as compared to the second phase. We found that those who received Androgel® in phase one were 78% ($1.68) less generous than participants on placebo (two-tailed t-test, p = .001). This indicates that participants became more generous during the course of the experiment. A similar effect was found on the rejection threshold; rejection was 30% ($0.88) higher (two-tailed t-test, p = .01) for participants getting T in the first phase compared to those given placebo. Across the two sessions, DM2s showed a lower likelihood of punishing stingy offers.

Because of these effects, we tested whether the parametric relationship between T, generosity and punishment maintained significance controlling for the order of Androgel® administration. Running a least squares regression on generosity, change in T values, only for those receiving Androgel® in the first phase, we found that men whose T was elevated due to Androgel® continued to be less generous (total T: β = −.44, p = 0.001; free T: β = −.05, p = 0.038; DHT: β = −.001, p = 0.009, N = 88). Nearly identical results obtain when those receiving placebo in the first stage are analyzed. We ran the same analysis for the rejection threshold and again found that punishment of those who were not generous increased with change in T levels (total T: β = .27, p = 0.007; free T: β = .03, p = 0.076; DHT: β = 0.017, p = 0.009, N = 88). Including the entire N = 200 data set in a least squares regression for generosity, the change in T (total, free, or DHT separately) and a binary order indicator again the negative relationship between T and generosity continues to be significant (free T; p = .003; total T: p = .001; DHT: p = .001; all two-tailed t-tests).

We also had participants make decisions in the DG in order to dissociate generosity and altruism [33]. Altruism is defined as giving to help another, while generosity is giving more than the other needs; the latter being a subset of the former. Altruism, as measured by offers in the DG, was not different for those on Androgel® compared to placebo (T: $3.34, Placebo: $3.56, two-tailed paired t-test, p = 0.86). No parametric relationship between DG offers and any measure of T was found. Because differences in altruism might impact generosity, we controlled for altruism and again examined the effect of testosterone on generosity in a least-squares regression. The parametric relationship between T levels and reduced generosity continued to maintain significance when DG offers were included (total T: β = −0.400, p = 0.001; free T: β = −0.057, p = 0.013; DHT: β = −0.001, p = 0.001).

Lastly, we analyzed the survey responses of participants to examine their affective states on and off Androgel®. Using paired two-tailed t-tests, p values for possible differences are: ECR-R (overall, p = .52; anxious attachment p = .55; avoidant attachment p = .81), IRI (p = .17), Anger (p = .84), PRI (p = .69). Participants on Androgel® were marginally more emotionally labile (AIM, p = .07). This indicates that temperament and mood were stable throughout the experiment.

## Discussion

Our primary finding is that manipulating T in men causes them to be 27% less generous in the UG then themselves at baseline. Interestingly, the threshold to initiate costly punishment for those who are less generous towards them increases with T levels. Indeed, participants on Androgel® were more than twice as likely to have exhibited negative generosity (rejection threshold exceeding proposed split) compared to themselves on placebo. This increase in negative generosity between conditions suggests that T infusion interfered with participants' ability to understand others' behaviors since rejections of DM1 proposals do not earn participants any money. These results are credible because T was directly manipulated, and the change in T was documented through blood draws. Further, the effects of T on generosity and punishment scale with a man's T levels, and the comparisons are within-subjects.

Our findings suggest that men with naturally high T levels would be expected to be more selfish and also more likely to punish others for violations of social norms, consistent with many correlational studies using retrospective reporting of behaviors and salivary T measures (11). Our results are not due to T making men more impulsive. A recent paper measuring salivary T finds that high T males were more patient in waiting for rewards that were promised in the future [39]. The parametric relationship we found between T levels, generosity, and punishment held whether men had their T levels manipulated or not. Because T responds to environmental conditions, our findings can provide insights about the origins of selfish and violent behaviors ranging from reckless driving, to watching or engaging in sporting events, to soldiers fighting in war. If rejections of stingy offers is an effort to punish violators of sharing norms at a cost to oneself, then a high rejection threshold can be considered a prosocial behavior at odds with the stinginess high T males exhibited in proposing splits in the UG. This may explain a variety of gender differences in seeking to enforce rules of conduct.

These findings can be compared to a study of generosity in the UG in which the neurohormone oxytocin (OT) was manipulated in men through intranasal infusion. In that study, those given 40 IU of OT were 80% more generous than participants on placebo, and no effect was found on the punishment threshold [33]. In a related study, males and females who were primed with an empathy-inducing video had a spike in plasma OT, and their generosity in the UG scaled positively with their subjective empathy ratings. This suggests that generosity is driven by feelings of empathy [40].

The opposite effects of T and OT on generosity may be caused by the interactive effects of these hormones. There is some evidence that T inhibits OT receptor binding [41], [42]. Giving T to females reduces empathy [43] and prenatal testosterone measures have been associated with inhibited affective and social descriptions at age four in boys [44]. By administering T, we may have inhibited OT binding and reduced empathy for the other person in the dyad. The impact of T on OT in the UG is, at this point, speculative.

T administration may have influenced the functioning of the hypothalamic-pituitary-adrenal (HPA) axis as there is significant cross-talk between these systems [45]. Our previous research showed that blood draws do not affect adrenocorticotropic hormone (ACTH) levels [46], and it is well-established in animals that T infusion inhibits ACTH release [47]. This suggests that the T administration increasing the punishment threshold is not due to HPA axis effects.

Related research used tryptophan depletion to reduce serotonin levels and then had participants make decisions as DM2s in the UG. Those with reduced serotonin rejected approximately 85% of highly unfair offers (20% of DM1 endowment) compared to an approximately 70% rejection rate for these offers for placebo participants. A similar finding using transcranial magnetic stimulation (TMS) to disrupt activity in the right prefrontal cortex found that rejections by DM2s in the UG of 20% of endowment offers fell to 85% from 91% compared to sham TMS [48]. Yet seven patients with ventromedial prefrontal lesions who played the UG as DM2s rejected 20% of endowment offers 74% of the time, while healthy controls rejected 50% of these [49].

What we have found is that T appears to play a role inducing men to change from being selfless to being selfish.
